# Metagenomic Evidence of Microbial Community Responsiveness to Phosphorus and Salinity Gradients in Seagrass Sediments

**DOI:** 10.3389/fmicb.2018.01703

**Published:** 2018-07-30

**Authors:** Matthew W. Fraser, Deirdre B. Gleeson, Pauline F. Grierson, Bonnie Laverock, Gary A. Kendrick

**Affiliations:** ^1^School of Biological Sciences, The University of Western Australia, Crawley, WA, Australia; ^2^Oceans Institute, The University of Western Australia, Crawley, WA, Australia; ^3^UWA School of Agriculture and Environment, The University of Western Australia, Crawley, WA, Australia; ^4^School of Science, Auckland University of Technology, Auckland, New Zealand

**Keywords:** phosphorus limitation, metagenomics, seagrass, phosphatase, organic phosphorus, planctomycetes, arylsulfatases, seagrass–bacteria interaction

## Abstract

Sediment microorganisms can have profound influence on productivity and functioning of marine ecosystems through their critical roles in regulating biogeochemical processes. However, the identity of sediment microorganisms that mediate organic matter turnover and nutrient cycling in seagrass sediments is only poorly understood. Here, we used metagenomic sequencing to investigate shifts in the structure and functioning of the microbial community of seagrass sediments across a salinity and phosphorus (P) availability gradient in Shark Bay, WA, Australia. This iconic ecosystem is oligotrophic and hypersaline with abundant seagrass meadows that directly contribute Shark Bay’s status as a World Heritage Site. We show that sediment phosphonate metabolism genes as well as enzyme activities increase in hypersaline conditions with lower soluble reactive phosphate in the water column. Given that sediment organic P content is also highest where P concentrations in the water column are low, we suggest that microbial processing of organic P can contribute to the P requirements of seagrasses at particularly oligotrophic sites. Seagrass meadows are often highly productive in oligotrophic waters, and our findings suggest that an increase in the functional capacity of microbial communities in seagrass sediments to break down organic P may contribute to the high productivity of seagrass meadows under oligotrophic conditions. When compared to soil and sediment metagenomes from other aquatic and terrestrial ecosystems, we also show microbial communities in seagrass sediments have a disproportionately high abundance of putative phosphorus and sulfur metabolism genes, which may have played an important evolutionary role in allowing these angiosperms to recolonize the marine environment and prosper under oligotrophic conditions.

## Introduction

Seagrass meadows are globally important ecosystems, providing habitat for many iconic marine organisms such as turtles and dugongs (Kendrick et al., 2012). These systems also sustain many important fisheries species (Gillanders, 2006), store significant amounts of “blue carbon” in sediments (Fourqurean et al., 2012a), and increase marine sedimentation rates by trapping particulates from the water column (Agawin and Duarte, 2002). The sediments (Welsh, 2000) and tissues (Garcias-Bonet et al., 2012) of seagrass meadows also present unique habitats for marine microorganisms. As the only marine angiosperms, seagrasses possess true root systems, which oxygenate surrounding rhizosphere sediments and create conditions that support high levels of bacterial diversity compared with adjacent unvegetated sediments (Garcia-Martínez et al., 2009). Microbial communities in seagrass sediments may in turn support seagrass productivity by increasing nutrient availability (Ikenaga et al., 2010) or oxidizing potentially toxic sulfides (Holmer et al., 2005). Studies examining microbial communities from seagrass sediments have primarily focused on their taxonomic structure (Jensen et al., 2007; Crump and Koch, 2008; Garcia-Martínez et al., 2009; Ikenaga et al., 2010), though the functional roles that microbial communities have in seagrass ecosystems is increasingly being recognized, particularly in impacting sulfur cycling through both sulfide oxidation and sulfate reduction under a range of environmental conditions (Ettinger et al., 2017a,b; Fahimipour et al., 2017; Trevathan-Tackett et al., 2017; Crump et al., 2018; Martin et al., 2018). However, the contribution that sediment microorganisms have in mediating phosphorus availability in seagrass sediments has not been addressed.

Microorganisms have an integral role in maintaining productivity of oligotrophic ecosystems in particular, whereby the abundance, diversity and expression of genes that promote efficient nutrient uptake and recycling increase as the availability of nutrients declines (Orchard and Webb, 2009; Shemi et al., 2016). Increased expression of key functional genes results in greater production of extracellular enzymes capable of degrading organic matter and altering nutrient cycling rates (Wardle et al., 2004; Orchard and Webb, 2009). For example, in the water column of the ultra-low P Sargasso Sea (<10 nmol L^-1^), bacteria and other microorganisms as well as some phytoplankton utilize polyphosphates as their primary P source; these microorganisms also have an increased capacity for storing and degrading these compounds relative to plankton and bacteria communities in nutrient rich waters (Martin et al., 2014). We might expect that sediment microbial communities in oligotrophic seagrass meadows also have an increased capacity to utilize novel P compounds relative to meadows in areas with higher phosphate availabilities.

The waters of Shark Bay are considered highly oligotrophic yet the Bay supports some of the largest seagrass meadows in the world. These meadows have been estimated to sequester around 243 Mg organic C ha^-1^ in sediments, making it a global hotspot for coastal organic C burial that is comparable with organic C stored in terrestrial forests (Fourqurean et al., 2012b). This enormous reservoir of sediment organic matter presents a potentially significant but largely uncharacterized nutrient supply that could help sustain the relatively high productivity of Shark Bay’s seagrass meadows, and alter biogeochemical processes and microbial community structure in oligotrophic sediments (Fraser et al., 2015).

The surface waters of Shark Bay are characterized by a natural salinity and P availability gradient; these are two key environmental drivers recognized globally that influence plant productivity and trophic structure in seagrass ecosystems (Walker, 1985; Deegan et al., 2002). Such environmental gradients are likely to modify the biogeochemical cycling of P and other nutrients through their influence on the structure of sediment microbial communities and key functional gene abundances (Fierer et al., 2012). Salinity rises from normal marine salinity (∼35‰) in the north of Shark Bay to over 65‰ in the southern and most hypersaline reaches, which also support Earth’s most extensive stromatolite populations (Kendrick et al., 2012). The seagrass meadows contribute to this salinity gradient by restricting water circulation across parts of Shark Bay (Kendrick et al., 2012). Soluble reactive phosphorus (SRP) – the most available source of P for biological uptake – decreases in the water column along this same gradient and is extremely low (<0.02 μM) in high salinity areas (Atkinson, 1987; Fraser et al., 2012). Total sediment P also decreases along the salinity gradient (Atkinson, 1987; Fraser et al., 2012), though the proportion of P present in organic forms in sediments increases (Fraser, 2016, Unpublished).

**FIGURE 1:**
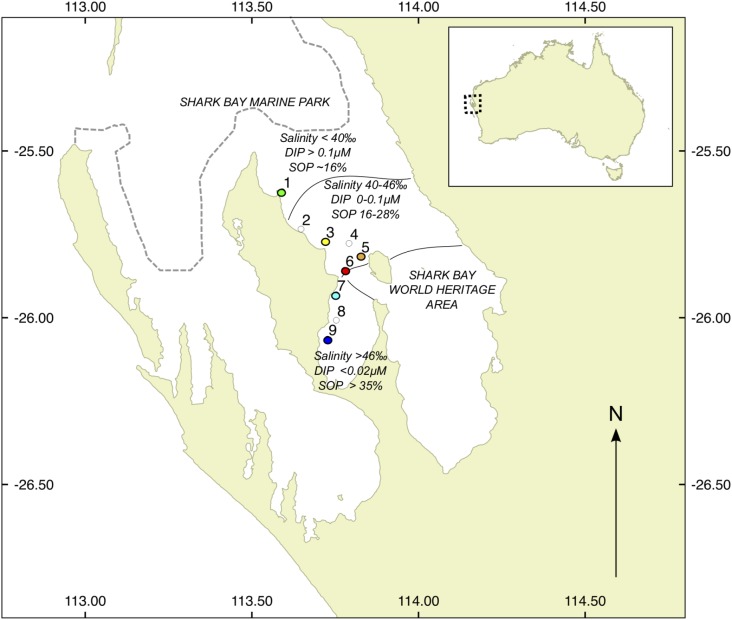
Location of study sites in Shark Bay, WA, Australia. Colored dots show sites where metagenomic profiles were generated, with colors corresponding to legends in later figures. White dots denote sites of additional samples from biogeochemical measurements. DIP, dissolved inorganic phosphate; SOP, sediment organic phosphorus.

Here, we used metagenomic sequencing to determine taxonomic and functional changes in the microbial communities of sediments from six seagrass meadows in Shark Bay (**Figure 1**) encompassing a range of salinities (39.2–53.2‰, **Supplementary Table S1**) and water SRP concentrations from 0.1 μM to below detectable concentrations (<0.02 μM). We also compared the microbial metagenomes of seagrass sediments from this study with other aquatic and terrestrial ecosystems to determine if the environmental conditions of seagrass sediments create uniquely adapted microbial communities. We hypothesized that: (i) the taxonomy and function of microbial communities in seagrass sediments would significantly change along the gradient; (ii) microbial communities at high salinity/low SRP sites would possess functions that are advantageous for P uptake in such environments (i.e., higher abundance of P metabolism genes); and (iii) microbial function in seagrass sediments would be correlated to enzyme activities in the sediments.

## Materials and Methods

### Sampling Sites and Experimental Design

Seagrass sediments and seagrass samples were collected from nine shallow (1.5–2.5 m) subtidal sites across Shark Bay, WA, Australia in March 2014. The seagrass communities at all sites were dominated by the temperate seagrass *Amphibolis antarctica*, with the tropical species *Halodule uninervis* as a sparse understory. The nine sites were chosen to encompass a range of salinities and SRP concentrations, from site 1 (lowest salinity, Guichenault Point) to site 9 (highest salinity, L’Haridon Bight) (**Figure 1**). All sites were separated by at least 5 km. At each site, four sediment samples (0–10 cm deep) were taken using 50 mL plastic syringe core, and transferred to 50 mL centrifuge tubes, for later biogeochemical and enzyme analysis. *A. antarctica* leaves (*n* = 100) were also hand-collected at each site for P content analysis. Samples were stored on ice in the field for no longer than 8 h, and subsequently stored at 4°C (sediment samples) or -20°C (seagrass leaves) until processing. In addition, five extra seagrass-associated sediment samples (0–5 cm) were collected using an ethanol-sterilized 3 mL syringe core, transferred to 3 mL cryovials and snap frozen immediately in liquid nitrogen for later metagenomic analysis. Cryovials were then stored at -80°C until DNA extraction. On return to the laboratory, seagrass leaves were thawed and epiphytes were removed from leaves by gentle scraping with a razor blade under deionised water. Leaves were dried at 60°C for 3 days, then ground using a mortar and pestle. P content on a subsample was determined using standard dry oxidation acid hydrolysis techniques (Solórzano and Sharp, 1980). SRP for water samples was measured colorimetrically on filtered (0.45 μm) water samples collected from each site using standard methods (Murphy and Riley, 1962).

### Enzymes

The activities of three hydrolytic enzymes were measured using standard colorimetric methods (Dick, 2011). The enzymes and substrates used were (i) β-glucosidase assayed with 4-nitrophenyl β-glucopyranoside, (ii) acid phosphatase, and (iii) alkaline phosphatase both assayed with 4-nitrophenyl phosphate disodium hexahydrate. For each assay, 1.00 g of sediment was added to a 50 mL Erlenmeyer flasks with 4 mL of modified universal buffer (pH = 6.00 for β-glucosidase, pH = 6.50 for acid phosphatase and pH = 11.00 for alkaline phosphatase) and 1 mL substrate, and then incubated at 37°C for 1 h. Following incubation, 1 mL of 0.5 M CaCl_2_ and 4 mL of 0.5 M NaOH (for phosphatases) or 4 mL 10 mM tris(hydroxymethyl) aminomethane (pH = 12, for glucosidases) were added to stop the reaction. The final nitrophenol concentrations were determined photometrically at 400 nm against a standard curve. Microbial P was operationally calculated as the difference in resin-extractable P between non-fumigated and hexanol-fumigated sediment samples using standard methodology (Kouno et al., 1995).

### Microbial Community Identification

A subset of six sites was selected for metagenomic analysis (sites 1, 3, 5, 6, 7, and 9) to encompass a range of salinities. DNA extraction from 500 mg of three replicate sediments per site was performed using PowerSoil DNA Isolation Kit (MoBio, Carlsbad, CA, United States) according to the manufacturer’s instructions. Where extractions yielded less than 200 ng of DNA, additional extractions were performed to ensure final concentrations of neat DNA were above 200 ng. In total, 18 DNA samples were sequenced and demultiplexed (Australian Genome Research Facility, Australia) on an Illumina MiSeq sequencer yielding a total of 23.53 Gb of data with at least 2 million sequence reads per sample (**Supplementary Table S2** and **Supplementary Figure S1** for rarefaction curves). All raw sequences have been uploaded to the NCBI Sequence Read Archive under submission number SUB3811707.

### Metagenomic Annotation and Statistical Analysis

We used a read-mapping approach to analyze the taxonomic and functional composition of microbial communities in seagrass sediments to avoid suppression of low-abundance species (Thomas et al., 2012) and to allow comparison to metagenomes from other biomes that were unassembled. Unassembled reads were annotated using the Metagenomics Rapid Annotation (MG-RAST) pipeline version 3.6 (Meyer et al., 2008). Quality control was performed on sequences in MG-RAST, including dereplication, ambiguous base filtering, quality filtering (Cox et al., 2010), and length filtering (50 bp). Taxonomic profiles were generated using Representative Hit Classification of the M5NR database to allow comparisons of multiple metagenomes (Meyer et al., 2008), and functional profiles were generated using Hierarchical Classification against the Subsystems database (Overbeek et al., 2005), with a minimum alignment length of 50 bp and *E*-value cutoff of *E* < 1 × 10^-5^ (Dinsdale et al., 2008; **Supplementary Table S2**).

To examine statistical differences in taxonomic and functional profiles across Shark Bay sediments, data were analyzed using the Statistical Analysis of Metagenomic Profiles (STAMP) package (Parks et al., 2014). Taxonomic and functional profiles were normalized in STAMP by sequencing depth using number of reads. PCA plots were generated using Euclidian distances between taxonomic and functional profiles. *P*-values for all tests were calculated using ANOVA for multiple test comparisons and Tukey–Kramer test for *post hoc* analysis. Storey’s false discovery rate (FDR) was used for multiple test corrections, given its higher power than other correction methods such as Benjamini-Hochberg FDR (Storey and Tibshirani, 2003; Storey and Taylor, 2004). Effect sizes (η^2^) are provided with *P*-values to show magnitude of shifts and give context to the biological significance of significant results (Storey and Taylor, 2004).

To compare microbial communities from seagrass sediments in this study to those from other ecosystems, we downloaded metagenomic profiles from a range of terrestrial, aquatic, and marine habitats publicly available on MG-RAST (full details **Supplementary Table S3**). Where possible, we chose profiles obtained with similar average sequence lengths (∼300 bp) and using Illumina as a sequencing platform, though this was not always possible. We limited our analyses to unassembled metagenomes, as these comprised the majority of available metagenomes at the time of analysis. Again, taxonomic profiles were generated using Representative Hit Classification of the M5NR database, and functional profiles were generated using Hierarchical Classification against the Subsystems database, with a minimum alignment length of 50 bp and *E*-value cutoff of *E* < 1 × 10^-5^ (Dinsdale et al., 2008). Profiles were combined and analyzed using STAMP, with *post hoc* tests using the Games-Howell test to account for the unequal sample sizes (Parks et al., 2014). Storey’s FDR was used for multiple test corrections.

Relationships between sediment enzyme activity, microbial P, sediment organic matter content, and microbial taxa and functions were analyzed using linear regression analysis against salinity. Prior to analysis, data were checked for normality and homogeneity of variances. Due to assumptions being violated, non-parametric tests (Kruskal–Wallis) were used to examine the relationships for seagrass P content. Statistical tests were analyzed using R version 3.1.1 (R Core Team, 2014) using the base program and the “ggplot2” package for graphics (Wickham, 2009). To assess the effects of environmental variables on microbial community function, a distance based linear model (DistLM) was used as some predictor variables were correlated with one another (Clarke and Gorley, 2006; Anderson et al., 2008). DistLM were applied using the software Primer +PERMANOVA v6.1.16 (Clarke and Gorley, 2006; Anderson et al., 2008), with stepwise regression as the selection procedure and corrected Akaike Information Criteria (AICc) as the selection criteria.

## Results and Discussion

**FIGURE 2:**
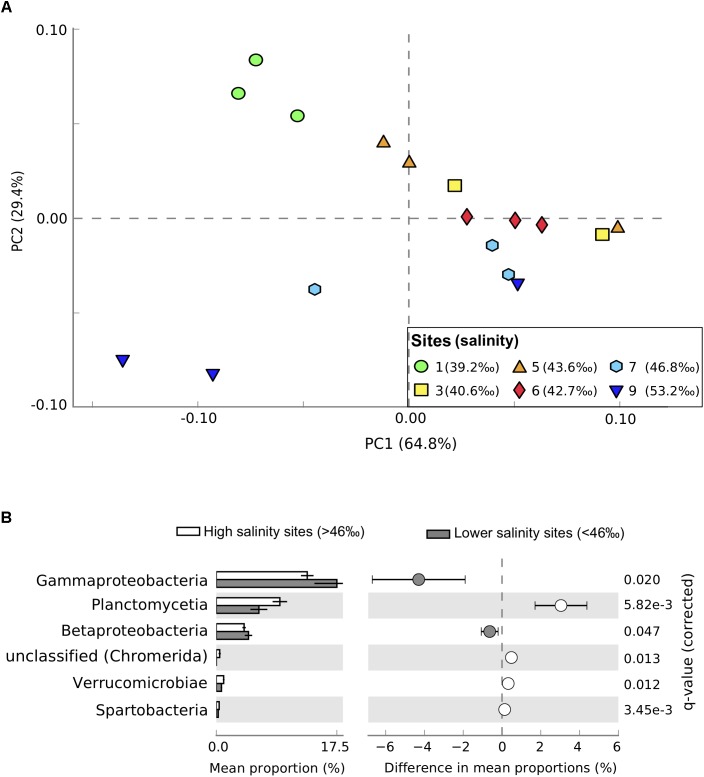
Taxonomic profiles from metagenomic sequencing of microbial communities in Shark Bay seagrass sediments. **(A)** Principal component analysis of taxonomic profiles between samples at the class level. Sites gradually increase in salinity from green (lowest salinity, circles, site 1) to dark blue (highest salinity, downward triangles, site 9). **(B)** Differences in relative abundance of classes between high salinity sites (>46‰, sites 7 and 9, white) and lower salinity sites (<46‰, gray). Only significantly different classes are shown, with corrected *P*-values calculated using Storey’s false discovery rate (FDR) approach (*P* < 0.05). Error bars show 95% confidence intervals.

We observed distinct shifts in the taxonomic and functional structure of seagrass-associated microbial communities across the salinity/P availability gradient. Proteobacteria were the dominant phyla in the seagrass sediment community, representing 48–53% of sequences. Other well represented phyla were Bacteroidetes (10–11%), Planctomycetes (6–9%), Firmicutes (5–6%), Actinobacteria (4.3–4.7%), and Cyanobacteria (3.6–5.9%). The most abundant classes detected were Deltaproteobacteria (15–17%), Alphaproteobacteria (11–14%), Gammaproteobacteria (13–19%), Planctomycetia (6–9%), Actinobacteria (4.3–4.7%), and Flavobacteriia (4.6–7.0%). Taxonomic profiles at sites >46‰ salinity separated from profiles generated from communities sampled at lower salinities along PC2 (**Figure 2A**). Proteobacteria were under-represented in the high salinity sites (sites 7 and 9, **Figure 2A**). Gene abundances for Betaproteobacteria (η^2^ = 0.64, *P* = 0.015, *n* = 18), Deltaproteobacteria (η^2^ = 0.57, *P* = 0.029, *n* = 18), Gammaproteobacteria (η^2^ = 0.78, *P* = 0.0023, *n* = 18), and Zetaproteobacteria (η^2^ = 0.88, *P* = 0.0002, *n* = 18) all significantly decreased along the salinity gradient. In contrast, Alphaproteobacteria tended to increase with increasing salinity (η^2^ = 0.53, *P* = 0.038, *n* = 18), as did Planctomycetes (η^2^ = 0.79, *P* = 0.005, *n* = 18) and Verrucomicrobia (η^2^ = 0.85, *P* = 0.001, *n* = 18), with highest abundances in sites 7 and 9 (**Figure 2B**). The class Cytophagia (phylum = Bacteroides) also increased significantly with salinity (η^2^ = 0.73, *P* = 0.0059, *n* = 18). Taxonomic shifts in microbial community composition was best explained by salinity, alkaline phosphatase expression, and temperature (AICc = 83.5, *R*^2^ = 0.57, **Supplementary Tables S4**, **S5**).

**FIGURE 3:**
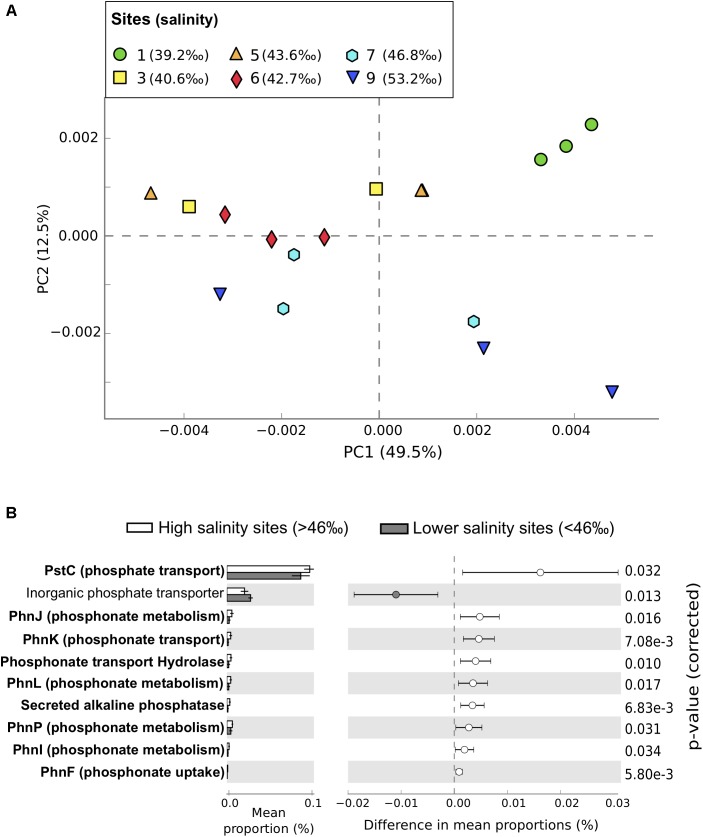
Functional profiles from metagenomic sequencing of microbial communities in Shark Bay seagrass sediments. **(A)** Principal component analysis of functional profiles between samples at the function level. Sites gradually increase in salinity from green (lowest salinity, circles, site 1) to dark blue (highest salinity, downward triangles site 9). **(B)** Differences in relative abundance of putative P metabolism functions annotated using the SEED Subsystems database between high salinity sites (>46‰, sites 7 and 9, white) and lower salinity sites (<46‰, gray). Functions related to phosphonate metabolism are in bold. Only putative P-metabolism functions that were significantly different between high and lower salinity sites are shown, with corrected *P*-values calculated using Storey’s FDR approach (*P* < 0.05). Error bars show 95% confidence intervals.

Functional gene abundances within the microbial community matched patterns in taxonomic composition, with sites >46‰ salinity differentiated from lower salinity sites along PC2 (**Figure 3A** and **Supplementary Figure S2**). Genes predicted to be related to microbial photosynthesis (η^2^ = 0.66, *P* = 0.0018, *n* = 18), P metabolism (η^2^ = 0.5, *P* = 0.018, *n* = 18), and DNA metabolism (η^2^ = 0.5, *P* = 0.022, *n* = 18) increased along the salinity gradient and were significantly more abundant in high salinity sites, while nitrogen (N) metabolism (η^2^ = 0.37, *P* = 0.039, *n* = 18) and cell signaling (η^2^ = 0.41, *P* = 0.024, *n* = 18) were significantly lower.

The gradient in P availability also contributed to significant changes in the capacity of microbial communities to utilize organic P compounds – in particular, phosphonates. Phosphonates are a significant component of the dissolved organic P pool in oceans, and in oligotrophic ecosystems can relieve P limitation for diazotrophs (N-fixing microorganisms) (Dyhrman et al., 2009). In our study, putative genes related to phosphonate degradation, transport, and metabolism were all significantly more abundant at high salinity sites where SRP concentrations decreased below detectable concentrations (**Figure 3B**). Putative genes for phospho acetaldehyde hydrolyse, important for phosphate release from phosphonates, also showed greater abundance at higher salinities (**Figure 3B**) suggesting an increased reliance of microorganisms on sediment organic P (SOP) within the high salinity sites. Given that the proportion of SOP increases in Shark Bay sediments with salinity (Fraser, 2016, Unpublished), microorganisms with the functional traits that allow them to use this resource would be at a competitive advantage. More broadly, the increase in abundance of putative genes related to breakdown of organic P compounds likely supports productivity in the most oligotrophic areas of Shark Bay through tight recycling of nutrients.

**FIGURE 4:**
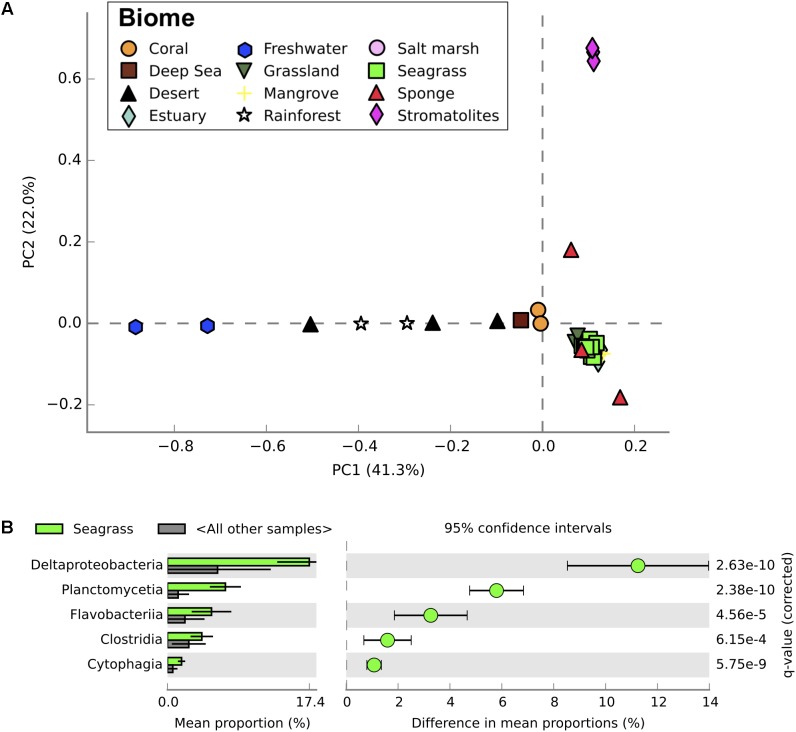
Taxonomic profiles from metagenomic sequencing of microbial communities from a range of different ecosystems. **(A)** Principal component analysis of taxonomic profiles (level = class) between microbial communities from all ecosystems examined. Seagrass communities from this study shown in bright green. **(B)** Relative abundance of classes between seagrass microbial communities and microbial communities from other ecosystems. Only significantly different classes are shown, with corrected *P*-values calculated using Storey’s FDR approach (*P* < 0.05). Error bars show 95% confidence intervals.

In addition to genes regulating the P cycle, the bacterial phylum Planctomycetes was more abundant within the high salinity compared to lower salinity sites of Shark Bay (**Figure 2B**). Planctomycetes was also more abundant in seagrass sediments of Shark Bay compared to metagenomic profiles from mangrove, saltmarsh, and a range of marine and terrestrial ecosystems (η^2^ = 0.848, *P* < 0.0001; **Figure 4**; **Supplementary Table S3**). While Planctomycetes have previously been found in other seagrass sediments (Jensen et al., 2007; Garcia-Martínez et al., 2009; Ikenaga et al., 2010; Cúcio et al., 2016) their specific interactions with seagrasses are unknown. Planctomycetes abundances may be high in seagrass sediments because of seagrass-derived organic exudates or litter (Fraser et al., 2015), providing an ideal niche where they can utilize a range of chemical compounds for energy, as has recently been observed in macroalgae (Lage and Bondoso, 2014). Seagrasses may also benefit from this association by making use of the nutrients released by Planctomycetes metabolic processes. Planctomycetes also produce abundant sulfatases, catalyzing the hydrolysis of organosulfates and impacting the S cycle (Wegner et al., 2013). Therefore, Planctomycetes may be key organisms mediating the turnover of organic matter the highly oligotrophic areas of Shark Bay, thus becoming increasingly important in maintaining productivity as P availability decreases.

Patterns in putative gene abundance do not always dictate actual expression of genes and subsequent biogeochemical shifts (Frias-Lopez et al., 2008). In this study, we found that putative gene abundance for secreted alkaline phosphatase significantly increased with salinity (*R*^2^ = 0.98, *P* < 0.0001, *n* = 18) and was also strongly correlated with extracellular activity of the alkaline phosphatase enzyme measured in sediments along the gradient (*R*^2^ = 0.88, *P* < 0.0001, *n* = 18). In agreement with previous studies (Fraser et al., 2012), there was, however, no corresponding increase in P content of seagrass leaves (**Supplementary Table S1**). Assuming enough substrate, phosphatase enzymes produced by bacteria would increase the availability of organic P to both microorganisms and seagrasses. We also found that putative genes related to sulfur (S) metabolism, including organic S metabolism and arylsulfatase, were more abundant in high salinity sediment and correlated with more abundant Planctomycetes. Arylsulfatase hydrolyses sulfur-ester bonds to release sulfate and is therefore crucial for sulfate reducers. However, arylsulfatases can also hydrolyze phosphate monoesters due to their non-specific nature, releasing phosphate (Luo et al., 2012). Given that phosphate monoesters are the dominant organic P compound in Shark Bay sediments (Fraser, 2016, Unpublished), the increase in sulfate reducers in high salinity sediments may indirectly increase P availability for seagrasses.

N fixation has previously been hypothesized as a major N source in hypersaline areas of Shark Bay, preventing N limitation (Atkinson, 1987). However, we found that genes predicted to be related to the nitrogen cycle (N fixation and nitrate/nitrite ammonification) were more abundant in sediments from low salinity/high SRP sites and less abundant in hypersaline areas. Nitrogen fixation in the water column and in sediments is energetically demanding and requires adequate concentrations of P (Sañudo-Wilhelmy et al., 2001). As such, microbial turnover of organic P may help overcome P limitation of N fixation in hypersaline Shark Bay sites, enabling productivity to be maintained in this extremely oligotrophic environment. Further research examining the abundance of diazotrophs in seagrass roots in oligotrophic ecosystems could show the ecological importance of endophytic bacteria in N acquisition under scenarios of nutrient limitation, and the requirement of adequate P cycling to supplement their capacity to fix N (Welsh, 2000; Garcias-Bonet et al., 2016).

**FIGURE 5:**
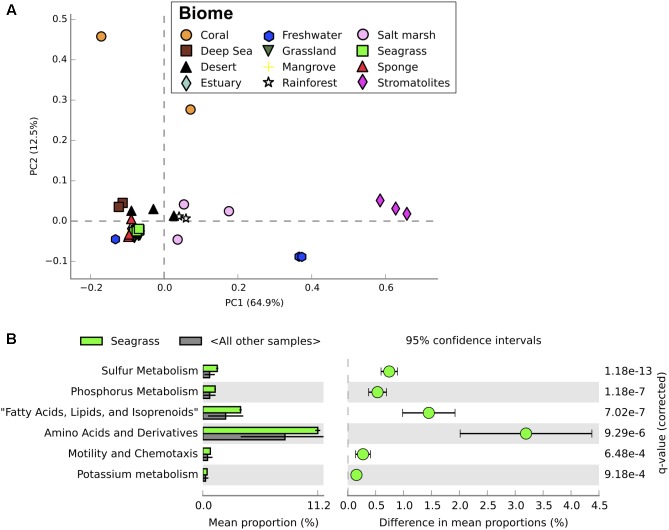
Functional profiles from metagenomic sequencing of microbial communities from a range of different ecosystems. **(A)** Principal component analysis of functional profiles (level 1 functions) between microbial communities from all ecosystems examined. Seagrass communities from this study shown in bright green. **(B)** Differences in relative abundance of putative functions between seagrass microbial communities and microbial communities from other ecosystems. Only significantly different functions are shown, with corrected *P*-values calculated using Storey’s FDR approach (*P* < 0.05). Error bars show 95% confidence intervals.

Seagrass-associated microbial communities were most similar to mangrove microbial communities when compared to metagenomic profiles from a range of different terrestrial and aquatic ecosystems (**Figures 4**, **5**). Mangroves and seagrass habitats share many environmental similarities; both are dominated by rooted macrophytes growing in organic-rich, anoxic sediments submerged in saline water. Microbial communities from other marine benthic environments, such as salt marsh and estuarine sediments, also showed taxonomic similarities to those from seagrass sediments (**Figure 4**). However, when comparing the functional profiles for the same communities, profiles for seagrass sediment communities showed greater overlap with functional profiles from mangrove, estuarine, and even some terrestrial ecosystems than with salt marshes (**Figure 5**). Microbial community structure in marine habitats can be driven by the composition of communities in adjacent habitats such as the surrounding water or sediments, with subsequent selection based on functional capabilities and environmental conditions (Burke et al., 2011). Seagrass sediments had significantly higher abundances of Deltaproteobacteria, Planctomycetes, and Flavobacteriia when compared with metagenomes from other ecosystems, and significantly lower abundances of Agaricomycetes and Dothideomycetes (**Figure 4**). Functionally, seagrass metagenomes had a high abundance of genes predicted to be related to sulfur and phosphorus metabolism (**Figure 5**), as well as amino acids and derivatives (**Supplementary Figure S3**). Previous amplicon sequencing showed the core rhizosphere microbiomes of the seagrasses *Zostera marina*, *Zostera noltii*, and *Cymodocea nodosa* were dominated by bacteria involved in the sulfur cycle (Cúcio et al., 2016), suggesting that these bacteria may be important in seagrass rhizospheres regardless of seagrass species or environmental conditions.

Microbial community composition can change selection pressure on plant traits, altering ecosystem processes (Lau and Lennon, 2011), while over evolutionary timescales have shaped the earth’s biogeochemical cycles through their impacts on pedogenesis in terrestrial ecosystems (Lambers et al., 2009). The specific adaptations within the microbial community point to potential co-evolution between seagrasses and sediment microorganisms that may help both groups thrive in the extreme, seemingly inhospitable environment of Shark Bay, characterized by high salinities, low nutrient availabilities, and high aridity. The relationship between sediment microorganisms and seagrasses is likely mutualistic, with seagrasses oxygenating and enriching sediments in organic matter, and microorganisms degrading organic matter and releasing nutrients for seagrass uptake that relieve P limitation. This appears critical for supporting productivity in this highly oligotrophic ecosystem. Similarly, recent studies investigating seagrass–microorganism interactions using culture-independent techniques have shown increased abundance of sulfate oxidizing bacteria around seagrass roots (Fahimipour et al., 2017; Martin et al., 2018), again pointing to a potentially symbiotic relationship. Further, seagrass microbial communities were taxonomically and functionally most similar to microbial communities from mangrove sediments (**Figures 4**, **5**). This suggests that specialized microbial communities are required in these marine sediments which are typically anoxic and enriched in organic matter, and that interactions between marine angiosperms and associated bacterial communities may have played an important evolutionary role in allowing terrestrial angiosperms to recolonize the marine environment.

## Data Accessibility

Full metagenomic profiles can be downloaded from MG-RAST under ID numbers 4661587.3–4661604.3 (**Supplementary Table S2**). Metagenomic profiles used for cross-ecosystem comparisons can also be accessed from MG-RAST using ID numbers in **Supplementary Table S3**. All raw sequences have been uploaded to the NCBI Sequence Read Archive under submission number SUB3811707.

## Author Contributions

MF, DG, PG, BL, and GK designed the study. MF, DG, and BL performed the research. All authors contributed to data analysis and writing the paper.

## Conflict of Interest Statement

The authors declare that the research was conducted in the absence of any commercial or financial relationships that could be construed as a potential conflict of interest.

## Supplementary Material

FIGURE S1 Rarefaction plot of taxonomic profiles generated from seagrass sediments across Shark Bay.

FIGURE S2 Changes in relative abundance of functional genes (annotated with SEED Subsystems database, level 2) between seagrass sediments in high salinity sites (>46‰, sites 7 and 9, white) and low salinity sites (<46‰, gray). Only putative functions that were significantly different between higher and lower salinity sites are shown, with corrected *P*-values calculated using Storey’s false discovery rate approach (*P* < 0.05). Error bars show 95% confidence intervals.

FIGURE S3 Changes in relative abundance of putative functions related to amino acids (annotated with SEED Subsystems database, level 3) between seagrass sediments (green) and microbial communities from other ecosystems (gray, **Supplementary Table S4**). Only putative functions that were significantly different are shown, with corrected *P*-values calculated using Storey’s false discovery rate approach (*P* < 0.05). Error bars show 95% confidence intervals.

TABLE S1 Summary of physical and biogeochemical data at each site. Depth, temperature, and salinity all averages over 5 CTD casts. Sediment organic matter, enzyme expressions, microbial P biomass, and seagrass P content are averages (*n* = 3 per site). DIP = dissolved inorganic phosphorus, BD = below detection.

TABLE S2 Summary of metagenomic sequencing results for Shark Bay sediments. Number of base pairs, sequencing reads, annotated proteins and % predicted using the SEED subsystem database after quality control on the MG-RAST pipeline.

TABLE S3 Summary of metagenomes used to compare microbial communities in seagrass rhizosphere sediments to other ecosystems. All metagenomes are publicly available on the MG-RAST server.

TABLE S4 DistLM results (marginal tests) of taxonomic community data (class level) against nine potential predictor variables (9999 premutations).

TABLE S5 DistLM results (Stepwise selection, sequential tests using corrected Akaike’s Information Criteria as selection criteria) of taxonomic community data (class level) against nine potential predictor variables (9999 premutations).
