# Supplementary figures and images for: Metagenomic Evidence of Microbial Community Responsiveness to Phosphorus and Salinity Gradients in Seagrass Sediments
Source: Front Microbiol. 2018 Jul 30;9:1703. doi: 10.3389/fmicb.2018.01703 (PMC6077243; doi:10.3389/fmicb.2018.01703)

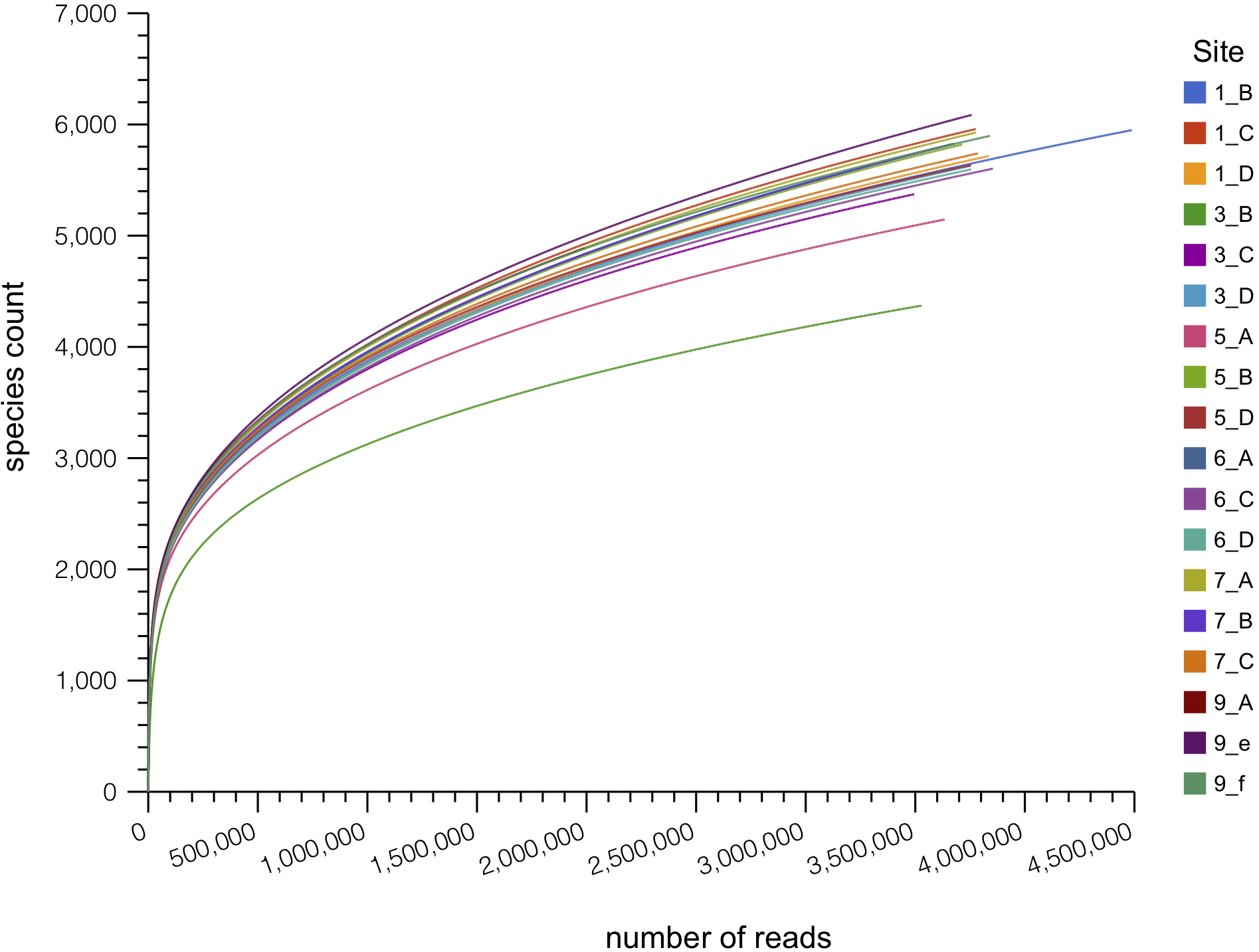

Supplement: FIGURE S1 — Rarefaction plot of taxonomic profiles generated from seagrass sediments across Shark Bay. [file Image_1.TIFF]

High Low

95% confidence intervals

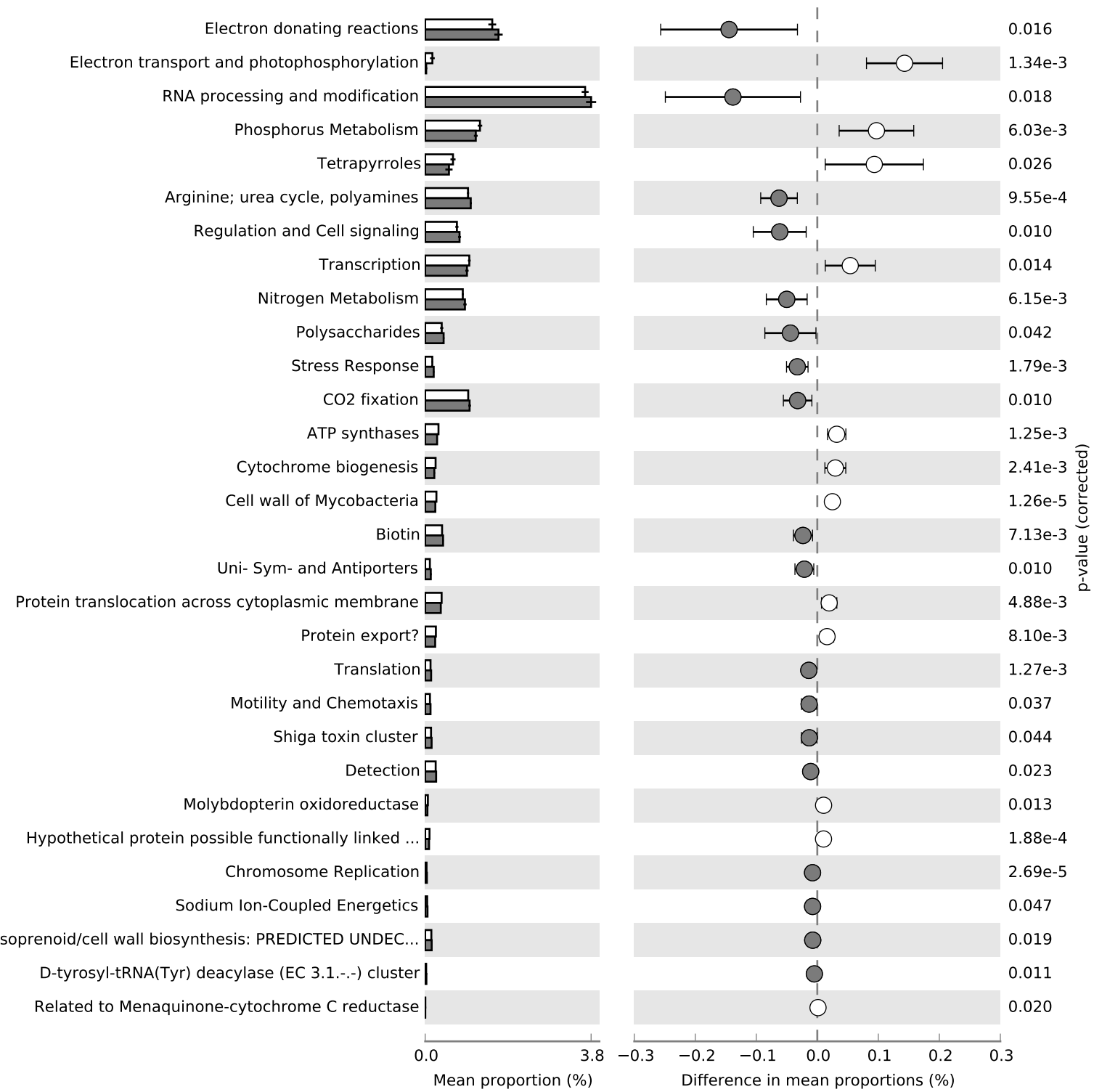

Supplement: FIGURE S2 — Changes in relative abundance of functional genes (annotated with SEED Subsystems database, level 2) between seagrass sediments in high salinity sites (>46‰, sites 7 and 9, white) and low salinity sites (<46‰, gray). Only putative functions that were significantly different between higher and lower salinity sites are shown, with corrected P-values calculated using Storey’s false discovery rate approach (P < 0.05). Error bars show 95% confidence intervals. [file Data_Sheet_1.PDF]

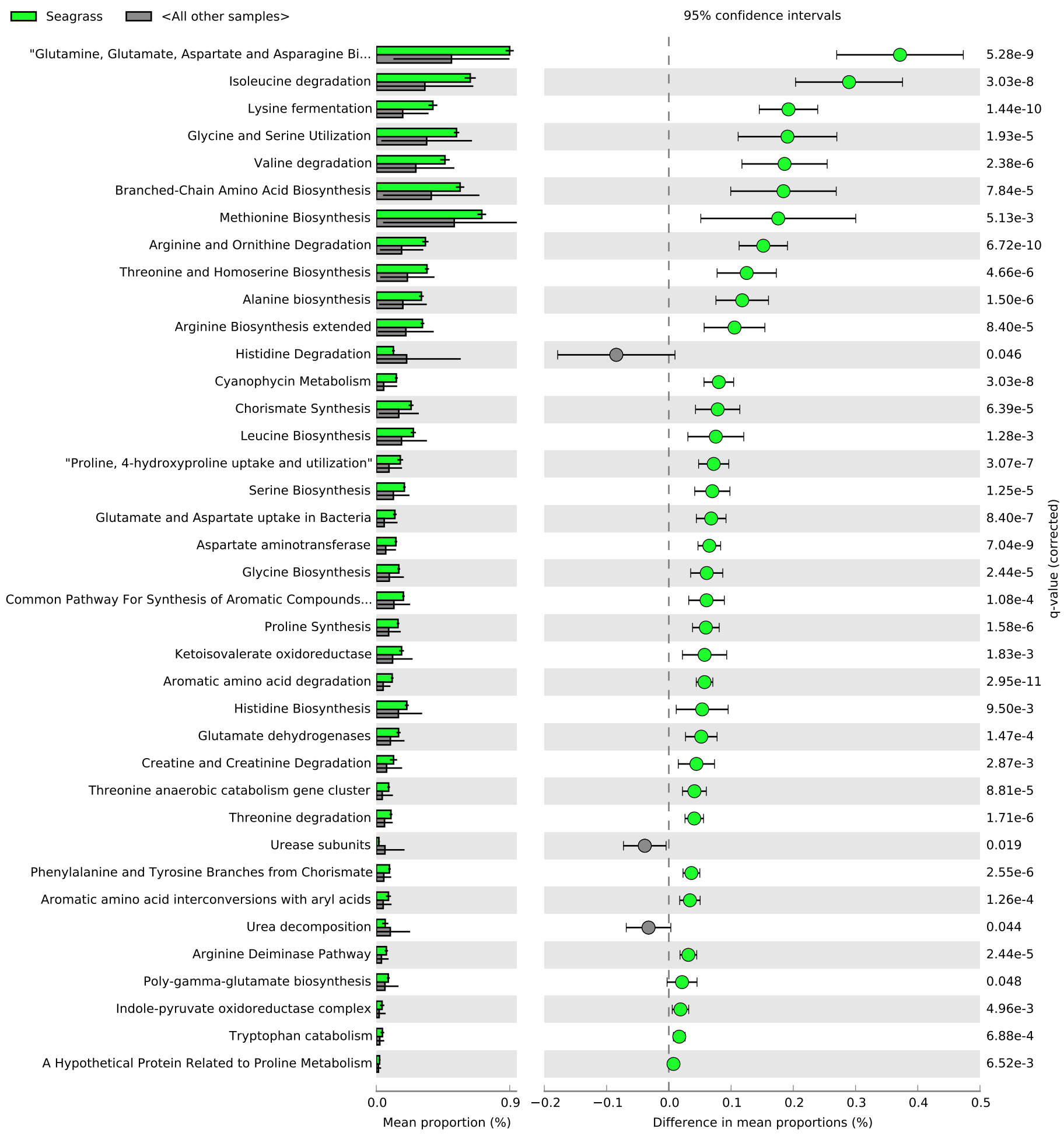

Supplement: FIGURE S3 — Changes in relative abundance of putative functions related to amino acids (annotated with SEED Subsystems database, level 3) between seagrass sediments (green) and microbial communities from other ecosystems (gray, Supplementary Table S4). Only putative functions that were significantly different are shown, with corrected P-values calculated using Storey’s false discovery rate approach (P < 0.05). Error bars show 95% confidence intervals. [file Data_Sheet_2.PDF]
