# Supplementary material for: Metagenomic Evidence of Microbial Community Responsiveness to Phosphorus and Salinity Gradients in Seagrass Sediments
Source: Front Microbiol. 2018 Jul 30;9:1703. doi: 10.3389/fmicb.2018.01703 (PMC6077243; doi:10.3389/fmicb.2018.01703)
Supplement: TABLE S1 — Summary of physical and biogeochemical data at each site. Depth, temperature, and salinity all averages over 5 CTD casts. Sediment organic matter, enzyme expressions, microbial P biomass, and seagrass P content are averages (n = 3 per site). DIP = dissolved inorganic phosphorus, BD = below detection. [file Table_1.DOCX]

**Table S1:** Summary of physical and biogeochemical data at each site. Depth, temperature, and salinity all averages over 5 CTD casts. Sediment organic matter, enzyme expressions, microbial P biomass, and seagrass P content are averages (n=3 per site). DIP = Dissolved inorganic phosphorus. BD = Below detection

| *Site* | *Depth (m)* | *Temp*  *(°C)* | *Water DIP*  *(µM)* | *Salinity (‰)* | *Organic matter*  *(% d.w)* | *Alkaline phosphatase (µmol^-1^ hr^-1^)* | *Acid phosphatase (µmol^-1^ hr^-1^)* | *Beta glucosidase (µmol^-1^ hr^-1^)* | *Microbial P biomass*  *(µg g^-1^)* | *Seagrass P (% d.w)* |
| --- | --- | --- | --- | --- | --- | --- | --- | --- | --- | --- |
| 1 | 1.8 | 25.7 | 0.1 | 39.2 | 1.7 | 123.9 | 40.4 | 4.7 | 35.0 | 0.06 |
| 2 | 1.6 | 26.3 | 0.1 | 39.7 | 2.7 | 31.0 | 23.9 | 4.9 | 16.0 | 0.05 |
| 3 | 1.9 | 26.4 | 0.06 | 40.6 | 1.5 | 26.5 | 17.1 | 3.4 | 8.1 | 0.05 |
| 4 | 1.3 | 25.0 | 0.08 | 42.3 | 4.3 | 102.2 | 43.3 | 8.5 | 7.3 | 0.05 |
| 5 | 1.5 | 24.4 | 0.06 | 43.6 | 2.0 | 66.8 | 39.3 | 4.7 | 15.5 | 0.06 |
| 6 | 1.6 | 26.0 | 0.05 | 42.7 | 4.1 | 131.0 | 71.0 | 17.4 | 52.3 | 0.07 |
| 7 | 1.5 | 26.1 | BD | 46.8 | 4.3 | 493.3 | 202.9 | 16.7 | 66.4 | 0.05 |
| 8 | 2.3 | 25.2 | BD | 51.2 | 2.1 | 239.0 | 79.3 | 3.1 | 54.7 | 0.06 |
| 9 | 1.8 | 25.3 | BD | 53.2 | 2.8 | 702.3 | 230.0 | 11.2 | 30.5 | 0.07 |

**Table S2:** Summary of metagenomic sequencing results for Shark Bay sediments. Number of base pairs, sequencing reads, annotated proteins and % predicted using the SEED subsystem database after quality control on the MG-RAST pipeline.

| *MG-RAST ID* | *Metagenome name* | *Site* | *Base pair count* | *Sequence count* | *Average sequence length* | *Annotated protein (%)* | *Number subsystem proteins* | *Predicted Subsystems proteins (%)* | *α-diversity*  *(species)* |
| --- | --- | --- | --- | --- | --- | --- | --- | --- | --- |
| 4661592.3 | MF1_B | 1 | 1,466,828,783 | 4,493,000 | 326 | 38.2 | 1,664,043 | 47 | 671 |
| 4661595.3 | MF1_C | 1 | 1,214,340,697 | 3,776,363 | 321 | 36 | 1,333,736 | 43 | 718 |
| 4661600.3 | MF1_D | 1 | 1,233,436,148 | 3,836,447 | 321 | 32.6 | 1,281,710 | 44.2 | 696 |
| 4661594.3 | MF3_B | 3 | 1,167,798,847 | 3,528,382 | 330 | 17.7 | 301,420 | 12.3 | 758 |
| 4661590.3 | MF3_C | 3 | 1,125,452,740 | 3,495,614 | 321 | 25.5 | 845,917 | 33 | 757 |
| 4661591.3 | MF3_D | 3 | 1,197,070,339 | 3,753,386 | 318 | 30.5 | 1,006,349 | 35 | 706 |
| 4661604.3 | MF5_A | 5 | 1,160,096,662 | 3,634,852 | 319 | 25.5 | 853,589 | 31 | 701 |
| 4661593.3 | MF5_B | 5 | 1,192,194,650 | 3,714,494 | 320 | 31.7 | 1,149,229 | 38 | 748 |
| 4661603.3 | MF5_D | 5 | 1,214,421,809 | 3,754,913 | 323 | 33.7 | 1,207,849 | 38 | 725 |
| 4661596.3 | MF6_A | 6 | 1,184,808,271 | 3,672,117 | 322 | 30.1 | 1,029,926 | 33 | 753 |
| 4661597.3 | MF6_C | 6 | 1,224,919,206 | 3,854,469 | 317 | 30.5 | 1,040,510 | 33 | 741 |
| 4661601.3 | MF6_D | 6 | 1,202,976,906 | 3,755,410 | 320 | 33 | 1,101,648 | 34 | 743 |
| 4661602.3 | MF7_A | 7 | 1,206,253,769 | 3,777,840 | 319 | 32.2 | 1,124,935 | 36 | 719 |
| 4661599.3 | MF7_B | 7 | 1,186,351,414 | 3,678,190 | 322 | 31 | 1,084,656 | 35 | 740 |
| 4661598.3 | MF7_C | 7 | 1,209,795,764 | 3,786,067 | 319 | 31 | 1,028,746 | 33 | 724 |
| 4661587.3 | MF9_A | 9 | 1,167,185,047 | 3,599,493 | 324 | 30 | 1,013,308 | 34 | 768 |
| 4661588.3 | MF9_e | 9 | 1,197,907,978 | 3,757,584 | 318 | 32.6 | 1,175,768 | 39 | 743 |
| 4661589.3 | MF9_f | 9 | 1,207,337,003 | 3,841,773 | 314 | 33.4 | 1,144,768 | 37 | 727 |

**Table S3:** Summary of metagenomes used to compare microbial communities in seagrass rhizosphere sediments to other ecosystems. All metagenomes are publicly available on the MG-RAST server.

| *Biome Type* | *Sample* | *MG-RAST IDs* | *Location* | *Sequencing method* | *Samples* |
| --- | --- | --- | --- | --- | --- |
| Seagrass (a) | Sediment | 4661587.3 – 4661604.3 | Shark Bay, Western Australia | Ilumina | 18 |
| Coral (b) | Tissue | 4445755.3 – 4445756.3 | Magnetic Island, Australia | 454 | 2 |
| Deep Sea (c) | Sediment | 4487294.3, 4487295.3 | Offshore sediment, South China Sea | Ilumina | 2 |
| Desert (d) | Soil | (i) 4477805.3,  (ii) 4477872.3-4477873.3 | (i) Mojhave Desert, California  (ii) Chihuahuan Desert, New Mexico | Ilumina | 3 |
| Estuary (e) | Sediment | 4440948.3, 4441020.3 - 4441022.3 | Coorong Lagoon, South Australia | 454 | 4 |
| Freshwater (f) | Sediment | 4465820.3, 4465821.3, 4465822.3 | Colorado River, USA | 454 | 3 |
| Grassland (g) | Soil | 4539064.3, 4541651.3 | Cedar Creek, USA | Ilumina | 2 |
| Mangroves (h) | Sediment | 4523017.3 – 4523020.3 | Red Sea, Saudi Arabia | 454 | 4 |
| Rainforest (i) | Soil | 4497403.3, 4497404.3 | Amazon Forest, Brazil | Ilumina | 2 |
| Salt marsh (j) | Sediment | 4520021.3, 4520022.3, 4520023.3 | Plum Island, USA | Ilumina | 3 |
| Sponge (k) | Tissue | 4530252.3, 4530290.3, 4530370.3 | Botany Bay, Australia | 454 | 3 |
| Stromatolite (l) | Microbial mat | 4604137.3, 4604139.3, 4604141.3 | Shark Bay, Western Australia | Ilumina | 3 |

(a) This study; (b) Littman *et al.* (2011) Environ Microbiolog Rep, 3:651; (c) Cai *et al.* (2013) *Applied Microbiology and Biotechnology* **97**:9579-9588; (d) Fierer *et al.* (2012) *PNAS* **109**:21390-21395; (e) Jeffries *et al.* (2012) *Biogeosciences* **9:**815-825; (f) Handley *et al.* (2014) *Environmental Microbiology* **16**:3443-3462; (g) Cline & Zak (2015) *Ecology* **96**:3374-3385; (h) Alzubaidy *et al.* (2016) *Gene* **576**:626-636; (i) Mendes (2014) Phd Thesis, University of Sao Paulo; (j) Graves *et al.* (2016) *Am Soc Microbiol* **82**:2862-2871; (k) Rua *et al.* (2015) *FEMS microbiology ecology* **91**:fiv043; (l) Wong *et al.* (2015) *Sci Rep* **5**:15607.

**Table S4:** DistLM results (marginal tests) of taxonomic community data (class level) against nine potential predictor variables (9999 premutations).

| *Variable* | *Pseudo-F* | *P-value* | *Proportion of variation* |
| --- | --- | --- | --- |
| **Salinity** | **4.3594** | **0.013** | **0.22518** |
| **Sediment alkaline phosphatase expression** | **3.7668** | **0.025** | **0.20072** |
| **Sediment acid phosphatase expression** | **3.6375** | **0.035** | **0.19517** |
| Sediment organic matter content | 2.3057 | 0.093 | 0.13323 |
| Sediment Beta glucosidase expression | 2.2046 | 0.105 | 0.12814 |
| Depth | 1.064 | 0.354 | 6.6235E-2 |
| Leaf seagrass phosphorus content | 0.90599 | 0.398 | 5.6959E-2 |
| Microbial P biomass | 0.51769 | 0.652 | 3.3361E-2 |
| Temperature | 0.40099 | 0.746 | 2.6037E-2 |

**Table S5:** DistLM results (Stepwise selection, sequential tests using corrected Akaike’s Information Criteria as selection criteria) of taxonomic community data (class level) against nine potential predictor variables (9999 premutations).

| *Variable* | *AICc* | *Pseudo-F* | *P-value* | *Percent variation explained* | *Cumulative frequency explained* |
| --- | --- | --- | --- | --- | --- |
| + Salinity | 87.163 | 4.3594 | 0.01 | 22.5 | 0.22518 |
| + Sediment alkaline phosphatase expression | 86.768 | 3.0833 | 0.056 | 13.9 | 0.36503 |
| + Temperature | 83.473 | 6.3731 | 0.011 | 20.9 | 0.57391 |
